# Automated Detection of Healthy and Diseased Aortae from Images Obtained by Contrast-Enhanced CT Scan

**DOI:** 10.1155/2013/107871

**Published:** 2013-03-31

**Authors:** Michael Gayhart, Hiroshi Arisawa

**Affiliations:** Department of Environment and Information Science, Yokohama National University, Yokohama 240-8501, Japan

## Abstract

*Purpose*. We developed the next stage of our computer assisted diagnosis (CAD) system to aid radiologists in evaluating CT images for aortic disease by removing innocuous images and highlighting signs of aortic disease. *Materials and Methods*. Segmented data of patient's contrast-enhanced CT scan was analyzed for aortic dissection and penetrating aortic ulcer (PAU). Aortic dissection was detected by checking for an abnormal shape of the aorta using edge oriented methods. PAU was recognized through abnormally high intensities with interest point operators. *Results*. The aortic dissection detection process had a sensitivity of 0.8218 and a specificity of 0.9907. The PAU detection process scored a sensitivity of 0.7587 and a specificity of 0.9700. *Conclusion*. The aortic dissection detection process and the PAU detection process were successful in removing innocuous images, but additional methods are necessary for improving recognition of images with aortic disease.

## 1. Introduction

Everyday quick and accurate decisions with inadequate information about a patient must be made by attending physicians at emergency departments. In 2007, in the United States, almost 6 million patients listed chest pains as their reason for visiting the emergency department [1]. Chest pain is a vague symptom which requires hospital admission or prolonged observation to determine the severity of a patient's condition. Negative results for inpatient cardiac evaluations cost around 6–8 billion dollars annually [2, 3]. Even with such precautions, within 6 months after a negative result, 2%–5% of these patients will have a serious cardiac event [4].

Over the past few decades, research into diagnostic radiology has provided more tools for handling symptoms of cardiac disease [5]. Due to recent improvements in multislice CT scanners, images taken in the cardiovascular region are clearer with less noise and artifacts and can be inspected with greater confidence [6, 7]. For diagnosing chest pain, several studies have demonstrated that a contrast-enhanced CT scan of thoracic cavity is an effective, accurate, and noninvasive method with a high negative predictive value for cardiac diseases [8–11]. One particular method is Triple Rule-Out (TRO) protocol in which the coronary arteries, pulmonary arteries, thoracic aorta, and other intrathoracic structures are highlighted in the CT scan. By examining the scan for signs of serious heart conditions, for instance coronary stenosis, pulmonary embolism, and aortic dissection (AD), TRO images can be used to determine if a patient should be released or admitted for further evaluation. This method results in the cutting of cost and time involved with diagnosing a patient. In recent years, several hospitals have implemented the TRO protocol along with other protocols of CT diagnosis as a part of their procedure for managing patients with vague symptoms at emergency departments.

As these protocols become widely used in diagnosing cardiac disease, the burden of a radiologist increases considerably. These methods require the time and abilities of well-trained radiologists to be utilized effectively. The number of cardiac images per patient produced during a CT scan depends on the thickness of each slice captured and can range anywhere from the hundreds to the thousands. During the diagnosis phase, actions may become repetitive due to the large number of images and many of the images have no signs of diseases. Moreover, there are difficulties in locating candidates of potential cardiac disease due to their small size or subtle appearance. In busy hospitals, evaluating these images may be overwhelming and this could lead to crucial misses in the diagnosis [12].

Therefore, we proposed and developed a computer aided diagnosis (CAD) system that could ease this burden of radiologists during the diagnosis of contrast-enhanced CT images of thoracic cavity. This would be accomplished by having the system perform two actions. First, identify innocuous images which would reduce the amount of time on repetitive actions by radiologists. Second, highlight areas of images with potential signs of cardiac disease which would reduce the chance of radiologists overlooking them. A successful automated diagnosis system would improve the promptness and accuracy of a radiologist's diagnosis.

Within data of a patient's CT scan is the possibility of many kinds of cardiac diseases. To develop methods for identifying all cardiac diseases in the CAD system would be a large undertaking. Therefore, we concentrate on one branch of cardiac disease, aortic diseases, where a CAD system could assist. For instance, an aortic dissection in the ascending aorta [13, 14] requires a careful observation to diagnose CT images and could be fatal if not recognized soon. There is little literature concerning CAD systems for aortic disease. Therefore, the focal point of this paper was to develop a diagnosis process for the CAD system to detect signs of aortic diseases within images produced by contrast-enhanced cardiac image scans.

In our previous research [15], a segmentation process was developed that finds and segments the pathway of an aortic artery known as the lumen. The segmentation method took into account all of these uncertainties properly, viewed the information from the whole image while allowing adjustments for local variations, and employed morphologic operators. The process provided our system with a clear blueprint of the shape, size, and boundaries of a patient's aortic lumen.

The data from the segmentation process provides the input for the diagnostic stage. The diagnostic process currently detects two aortic diseases, aortic dissection and penetrating aortic ulcer (PAU), in contrast-enhanced CT images of the aorta. Two criteria for signs of these diseases were established for the system to be utilized in its decision making process:

- for aortic dissection, if the cross-section of the aorta in an image is not circle-like;
- for PAU, if the aorta contains objects with HU values higher than normal along its wall.

**Figure 1:**
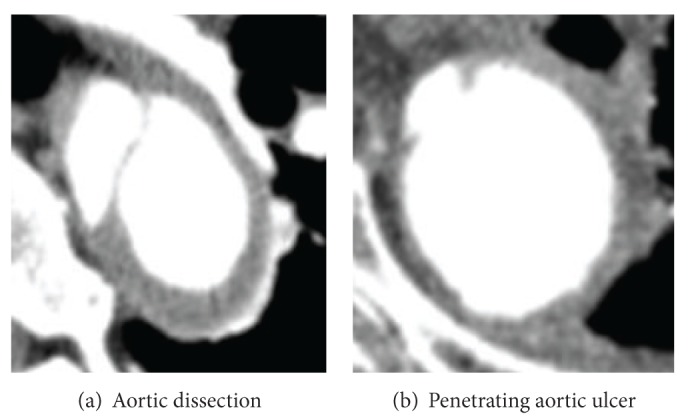
Examples of aortic disease.

In the case of an aortic dissection, blood flows into the media layer of a deteriorated section of the aortic wall and creates a new lumen. From the perspective of a CT image, the lumen is separated into two pieces and the differences in blood pressure between the two lumens cause distortions in their shapes (Figure 1(a)). The functions of the diagnostic process were designed based on edge oriented methods and should be capable of recognizing a circle-like object in an image. In the output, this process identifies aortic objects with a circle-like shape as healthy and any aortic object that does not meet that criterion as a possible candidate for aortic disease.

In the case of PAU, an ulceration of an atheromatous plaque erodes the intima causing a hematoma in the media of the aorta. In contrast-enhanced CT slices, a PAU appears as a contrast-filled, pouch-like protrusion of the aorta (Figure 1(b)) or as a thickened aortic wall in absence of an intimal flap or a false lumen. The Hounsfield value (HU) around the PAU can be higher than the normal lumen for two reasons. First, contrast media become temporarily trapped in these pouches and increase its concentration in this area. Second, the calcified plaque that causes PAU has a high HU value and lines the intima around the PAU. The functions for the diagnostic process were created using interest point operators to locate these objects of higher than average intensity within an aortic object. In the output every aortic object that meets this criterion is labeled as a PAU object and sign of a PAU. Otherwise, the aortic object as a whole is labeled as healthy.

To summarize, we built an automated process that determines whether an aortic object in a slice is a candidate for aortic dissection or PAU based on contrast-enhanced CT data. We report technical details of this method for this automatic identification and present preliminary results in applying the process to 9 cases of CT data.

## 2. Materials and Methods

### 2.1. Patient Selection

The patient data used in this study were obtained from Yokohama City University as a part of our laboratories joint research. Patients were undergoing an examination using the TRO protocol. They gave their permission for the use of their data in this study.

### 2.2. CT Technique

The basic procedure for TRO protocol used for this project, as developed at Yokohama City University, consisted of three phases: a scanogram, a precontrast scan, and a postcontrast scan.

All images were acquired with a 64-slice CT scanner. Scans were between the diaphragm and the top of the aortic arch.

The scanogram was a quick preliminary scan which determined the region where the precontrast and postcontrast scan will take place.

The precontrast scan was a full CT scan of the cardiac region with no injection of a contrast medium, a radioactive dye.

The postcontrast scan was a full CT scan of the cardiac region in which the patient was injected with contrast medium to illuminate key areas in the cardiac region. In this phase, the patient received 70 mL of contrast medium into the right arm to opacify the coronary arteries and the aorta and, a minute later, another 30 mL of contrast medium to opacify the pulmonary arteries. Once the contrast medium had reached a predetermined level in the blood stream, the post contrast scan began. The scan took on average about 15 seconds.

The data was stored in DICOM format, and the resolution for the images was 512 × 512 pixels. The diagnosis of the cases in this study is represented in Table 1.

### 2.3. Principles of the Segmentation Process

**Figure 2:**
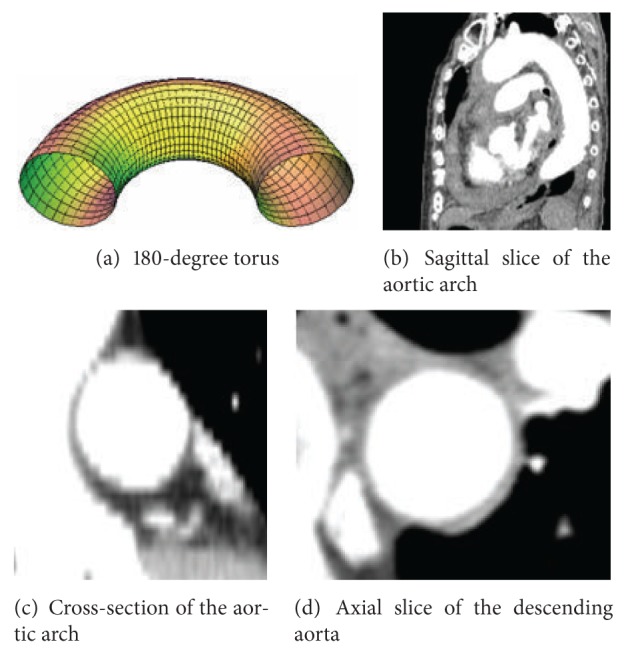
Criteria for segmentation process.

**Figure 3:**
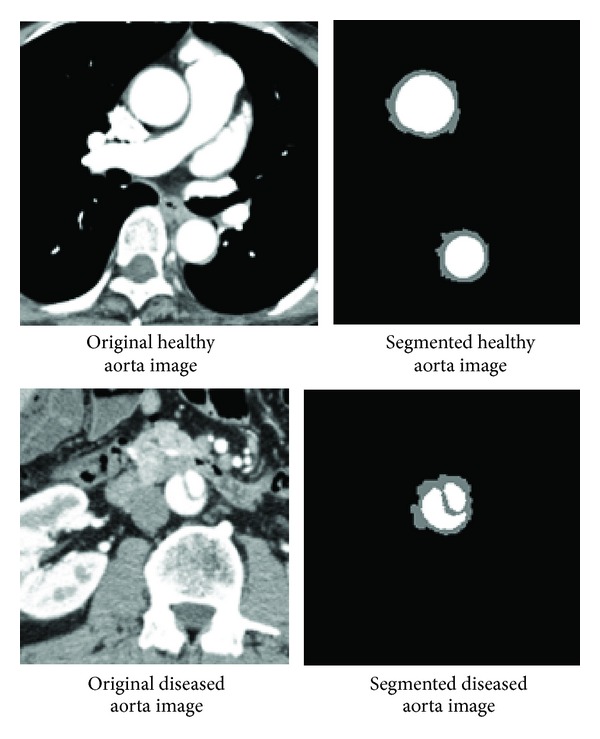
Results of segmentation.

The CAD system for the aortic artery in this study is designed to work with the data received from a CT scan, which is composed of three inputs: (1) the DICOM data of a patient's CT scan which includes cardiac images from the top of the aortic arch to the diaphragm, (2) the range of Hounsfield unit (HU) of the contrast-enhanced aortic lumen detected during the CT scan (average range 200 HU–500 HU), and (3) the starting positions of the ascending aorta and descending aorta. The two criteria for this process are as follows: (1) a cross-section of the aortic artery is circle like in shape (Figures 2(c) and 2(d)); (2) the aortic arch creates 180-degree torus (Figures 2(a) and 2(b)). From these inputs and criteria, the segmentation process identifies objects in the CT scan which are the aorta (Figure 3) and links them together and labels them as ascending aorta, descending aorta, or aortic arch depending on their location. Afterwards, the automatic segmentation outputs image sets of the ascending aorta, the descending aorta, and the aortic arch.

### 2.4. Principles of the Diagnostic Process for Aortic Dissection

**Figure 4:**
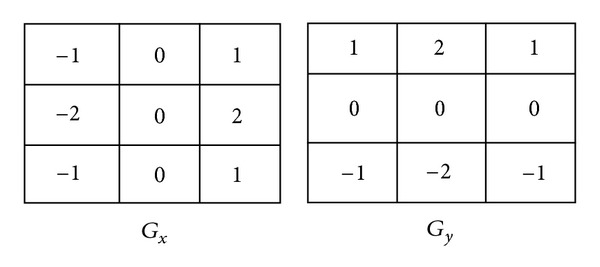
Convolution mask for the gradient.

Utilizing the segmentation data, the CAD system initializes the diagnostic process for aortic dissection by collecting two pieces of information. First, Sobel operator provides the diagnostic process with an approximation of the gradient image of segmentation data. The gradient values represent the degree of change of intensity in the *x*-and *y*-directions at particular pixel in an image, *G*_*x*_ and *G*_*y*_, respectively. A pair of convolution masks (Figure 4) is applied to the HU value of each pixel to calculate *G*_*x*_ and *G*_*y*_. Then, the gradient angle (*Gθ*) of each pixel is assessed with the following function:

(1) $$\begin{array}{c} G\theta =\tan^{-1}\frac{G_{y}}{G_{x}}. \end{array}$$

Second, the centroid (*C*_*x*_, *C*_*y*_) of an aortic object is determined. *n* is the total number of pixels in an object. *x*_*n*_  and *y*_*n*_ are the *x*-and *y*-positions of an individual pixel:

(2) $$\begin{array}{c} C_{x}=\frac{x_{1}+x_{2}+\cdots +x_{n}}{n},\\ C_{y}=\frac{y_{1}+y_{2}+\cdots +y_{n}}{n}. \end{array}$$

The Fast Circle Detection algorithm [16] examines the boundary of an object and determines if this object is a circle based on edge oriented methods. The decision is based on the number of unique boundary pixel pairs (*P*_1_, *P*_2_) that satisfy three conditions.

**Figure 5:**
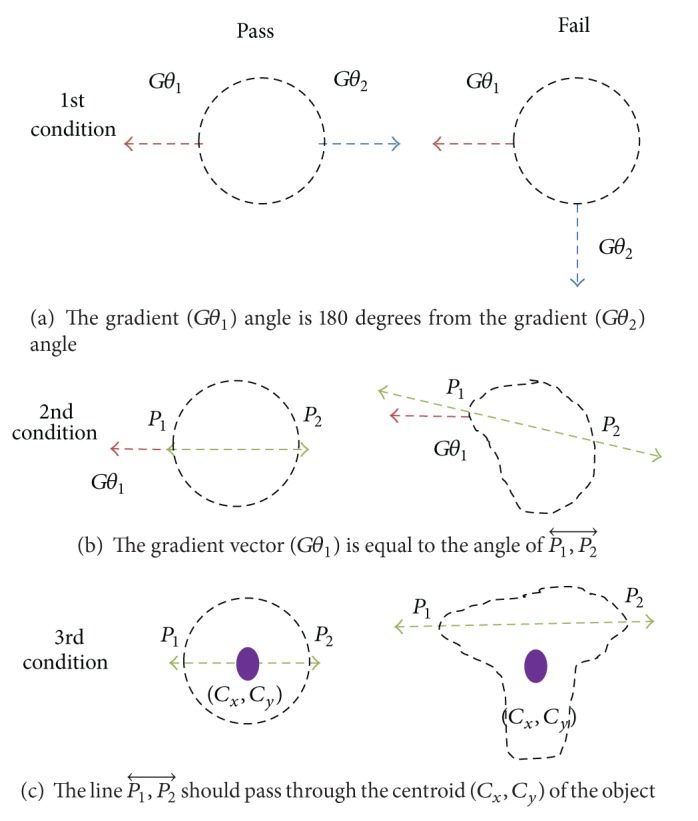
Fast Circle-Detection algorithm conditions.

(1) The gradient (*Gθ*_1_) angle of *P*_1_ is 180 degrees from the gradient angle (*Gθ*_2_) of *P*_2_ (Figure 5(a)):
  (3) $$\begin{array}{c} 180^{{}^{\circ}}=\left|{G\theta }_{1}-{G\theta }_{2}\right|. \end{array}$$
(2) The gradient vector (*Gθ*_1_) is equal to the angle of $\overset{⃡}{P_{1},P_{2}}$ (Figure 5(b)):
  (4) $$\begin{array}{c} {G\theta }_{1}=∠\overset{⃡}{P_{1},P_{2}}. \end{array}$$
(3) The line $\overset{⃡}{P_{1},P_{2}}$ should pass through the centroid (*C*_*x*_, *C*_*y*_) of the object (Figure 5(c)).

The percentage of boundary pixels that meet these conditions is calculated. If the percentage reaches the predetermined threshold, the object is labeled circle like and possibly healthy. If the percentage fails the threshold, the object is labeled abnormal shape and a candidate for aortic dissection.

### 2.5. Principles of the Diagnostic Process for PAU

**Figure 6:**
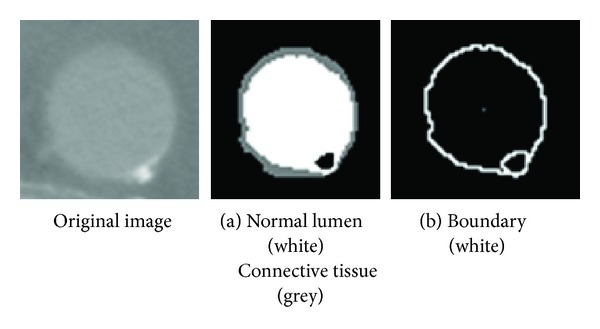
Detection of PAU.

The PAU detection process begins by analyzing the segmentation data of contrast media enhanced CT images. During the segmentation process, PAU and calcifications with strong intensities are removed from the image as their HU values are much higher than the normal lumen's intensity (Figure 6(a)). After the segmentation process, these PAU and calcifications appear as small ovals and ellipses around the edge of aorta (Figure 6(b)).

The PAU detection process is a search for pixels that meet these requirements:

1. this pixel's HU value is higher than the highest value of the range of the lumen's intensity;
2. this pixel is contained inside the boundary of an aortic object.

Through connected-component labeling, these pixels are grouped in PAU objects. These objects are highlighted in the image and output as a candidate for PAU.

### 2.6. Evaluation of Diagnostic Processes

The processes for detecting healthy aortas and candidates for diseased aorta were evaluated in two parts with 9 cases where the aortic region has been enhanced with contrast media in a CT scan. For this experiment, the system only examined the slices of the ascending aorta and descending aorta because their cross-section is readily viewable in the CT images without reconstruction. A cross-section of the aortic arch requires multiplanar reconstruction of the CT images which was not available at the time of testing.

The first part examined whether the Fast Circle Detection algorithm could correctly identify healthy and aortic dissection images in 5 cases which included 3 healthy and 2 aortic dissections. The system returned a decision of whether a slice of the aorta was healthy or a candidate for aortic dissection. The threshold for the Fast Circle Detection algorithm was set at 60% for the percentage of boundary pixels with a unique gradient pair out of the total number of boundary pixel in an object. The results were compared to the actual number of healthy aortic slices and candidate slices.

A true positive is defined as an aortic object that contains an aortic dissection and was labeled by the CAD system as a candidate for aortic dissection. A false positive is defined as an aortic object that is healthy but was labeled by the CAD system as a candidate for aortic dissection. A true negative is defined as an aortic object that is healthy and was labeled by the CAD system as healthy. A false negative is defined as an aortic object that contains an aortic dissection but was labeled by the CAD system as healthy.

The second part reviewed the process for detecting signs of PAU in the aorta with the 4 cases of PAUs. The system returned objects in a slice which were labeled as possible signs of PAU. The results were compared to the actual number of PAUs in set of test data. A true positive is defined as an aortic object that contains a PAU, and a candidate for PAU was detected by the CAD system. A false positive is defined as an aortic object that has no PAU, but a candidate for PAU was detected by the CAD system. A true negative is defined as an aortic object that has no PAU, and no candidate for PAU was detected by the CAD system. A false negative is defined as aortic object that contains a PAU, but no candidate for PAU was detected by the CAD system.

The system examined the images on a computer with Intel Core 2 Extreme CPU Q6850 3.00 GHz 2.99 GHz and 8 Gb of Ram. The total time of the process was also recorded.

## 3. Results

### 3.1. Results of the Diagnostic Process for Aortic Dissection

Of the 479 images from 5 cases, 161 slices contained the ascending aorta and 479 slices contained the descending aorta. The results are represented in Table 2.

Regarding the descending aorta, 83 were correctly identified as candidates for aortic dissection (true positive) and 0 were incorrectly identified as candidates for aortic dissection (false positive), 378 slices were correctly identified as healthy (true negative), and 18 were incorrectly identified as healthy (false negative). The false negative slices occurred when the aortic dissection only caused a change in the size of aorta but not the shape.

Concerning the ascending aorta, there was no occurrence of aortic dissection. 156 slices were correctly identified as healthy, and 5 slices were incorrectly identified as a candidate for aortic dissection. The false positive slices occurred where the ascending aorta was transitioning to the aortic arch.

### 3.2. Results of the Diagnostic Process for PAU

Of the 461 slices in the 4 cases with PAU, 213 slices contained the ascending aorta and 461 slices contained the descending aorta. 26 of the 213 slices containing ascending aorta were removed because of an error with the segmentation process, making the final total of slices of the ascending aorta 187. The results are represented in Table 3.

In the ascending aorta, 48 slices were correctly identified with PAU (true positive), 5 slices were incorrectly identified with PAU (false positive), 116 slices were correctly identified with no PAU (true negative), and 18 slices were incorrectly identified with no PAU (false negative).

In the descending aorta, 191 slices were correctly identified with PAU, 5 slices were incorrectly identified with PAU, 207 slices were correctly identified with no PAU, and 58 slices were incorrectly identified with no PAU.

False positives occurred when the contrast media were slightly above the intensity range of the lumen, most likely due to an artifact in the CT image. False negatives generally happened when the PAU's intensity was similar to the normal lumen's intensity.

### 3.3. Runtimes of the CAD System

The runtimes for the CAD system to segment and perform the diagnostic process explained in this paper are represented in Table 4.

## 4. Conclusion

With the purpose of designing a CAD system for aortic diseases to assist radiologists, we developed two automated diagnostic processes for determining whether an aortic object in a contrast-enhanced CT image contains candidates for aortic dissection or PAU. The results of our study have brought us the following conclusions about the feasibility of this system.

The aortic dissection detection process had a sensitivity of 0.8218 and a specificity of 0.9907. This indicates that this process is capable of identifying a healthy aorta shape but not fully able to recognize all types created by an aorta dissection. The most common error occurred when the aorta dissection caused the aorta to shrink but still maintained a circle-like shape. Additional methods must be utilized by aortic dissection process to collect relevant data, such as reduction in size and the appearance of an intimal flap in the aortic object, to reduce the false negatives.

The PAU detection process scored a sensitivity of 0.7587 and a specificity of 0.9700. This process mostly avoided incorrect identification of PAU. This is likely due to the success of this segmentation process with removing artifacts and noise. Regarding the sensitivity, this process was able to distinguish a majority of the PAU. When it overlooked a PAU, this was because the PAU had an intensity that was similar to the lumen. A supplementary method for recognizing the shape of PAU on the boundary of an aorta should be implemented to red9ce the false negatives.

This research will serve as a base for future studies of CAD system for aortic disease and expand on various methods for automated diagnosis. There were some limitations that should be addressed in the next study. The current study has examined two methods for identifying aorta dissection and PAU. More methods must be included to enhance the accuracy and precision of this CAD system. Moreover, the experimental data was relatively small. A larger set of test data is necessary for more definitive results.

In the future, concerning the aortic disease diagnosis process, more algorithms that address issues of image processing will be designed to analyze the aorta and to search for signs of aortic diseases such as aortic dissection, intramural hematoma, and penetrating atherosclerotic ulcer [13, 14]. Criteria still unaddressed for identifying aortic diseases include (1) significant changes in the size (increasing or decreasing) of aorta, (2) appearance of intimal flaps which are dark lines contained within the lumen, and (3) detection of PAU on the boundary based on shape.

In summary, the aortic dissection detection process and the PAU detection process were successful in removing innocuous images, but additional methods are necessary for improving recognition of images with aortic disease. These methods can be used for the next step in building a CAD system for detecting aortic diseases.

## Figures and Tables

**Table 1:** List of case data used in evaluation.

Case number	Condition	Number of slices
1	Healthy	62
2	Aortic dissection	25
3	PAU	129
4	Normal	186
5	PAU	189
6	PAU	182
7	Normal	202
8	PAU	112
9	Aortic dissection	125

**Table 2:** Results of the diagnostic process for aortic dissection.

Data type	True positive	False positive	True negative	False negative
All aorta data	83	5	534	18
Ascending data	0	5	156	0
Descending data	83	0	378	18

Sensitivity	0.8218		Specificity	0.9907

**Table 3:** Results of the diagnostic process for PAU.

Data type	True positive	False positive	True negative	False negative
All aorta data	239	10	323	76
Ascending data	48	5	116	18
Descending data	191	5	207	58

Sensitivity	0.7587		Specificity	0.9700

**Table 4:** Runtimes of the CAD system.

Case number	Images used	Time (min:sec)
1	62	2:24
2	25	0:55
3	129	5:08
4	186	10:32
5	189	10:58
6	182	10:06
7	202	12:20
8	112	4:45
9	125	5:17
